# Predictive value of surface electrocardiogram in localizing right ventricular outflow tract premature beats: a study combining intracardiac echocardiography and electroanatomical mapping under all-zero fluoroscopy ablation

**DOI:** 10.3389/fphys.2025.1610974

**Published:** 2025-06-19

**Authors:** Xiaoran Cui, Ruibin Li, Yichen Li, Yi Zhang, Jidong Zhang

**Affiliations:** Department of Cardiology, The Second Hospital of Hebei Medical University, Shijiazhuang, China

**Keywords:** premature ventricular contractions (PVCs), radiofrequency ablation, three-dimensional mapping, intracardiac echocardiography, right ventricular outflow tract (RVOT)

## Abstract

**Objective:**

This study aims to evaluate how well surface electrocardiograms (ECG) predict premature ventricular contraction (PVCs) originating from the right ventricular outflow tract (RVOT) and to examine the role of intracardiac echocardiography (ICE) during their radiofrequency ablation.

**Methods:**

From October 2022 to December 2023, we conducted a prospective study at the Second Hospital of Hebei Medical University, enrolling 20 consecutive patients with RVOT-PVCs. In our study, utilizing ICE and the CARTO 3D electroanatomical mapping system, all procedures were performed under a completely fluoroless condition. Ablation sites were categorized into two subgroups: supra-pulmonary cusp (n = 3) and sub-pulmonary cusp (n = 17). Preoperative ECG parameters were systematically analyzed. ICE and CARTO mapping were employed to precisely localize and ablate the PVC foci, with detailed spatial electroanatomical characterization.

**Results:**

Of the 20 patients, the origins of PVCs were found in two regions: 17 patients (85%) had PVCs originating from the sub-pulmonary cusp, while 3 patients (15%) had PVCs from the supra-pulmonary cusp. All supra-pulmonary cusp ablation targets were located in the left pulmonary cusp, 3.33 ± 0.33 mm above the cusp base. Sub-pulmonary cusp PVCs were predominantly clustered at the left anterior cusp junction (13/17, 76.5%), with one case (5.9%) at the right anterior cusp junction, averaging 2.53 ± 0.38 mm from the cusp base. Additionally, an R-wave duration ≥50 ms in lead V2 predicted sub-pulmonary cusp localization with 70.6% sensitivity and 100% specificity.

**Conclusion:**

ICE can clearly visualize the anatomical structures of the right ventricular outflow tract, which aids in accurately determining the ablation site. In lead V2, an R wave duration of 50 milliseconds or longer exhibits certain predictive value for ventricular premature beats originating below the pulmonary valve sinus. For PVCs originating below the pulmonary valve, the ablation target sites are primarily concentrated at the junction of the left-anterior pulmonary sinus cusp.

## Introduction

Ventricular premature beats, also known as premature ventricular contractions, occur due to early depolarization of ectopic excitation points in the ventricular muscle located below the His bundle and its branches. They are among the most common arrhythmias encountered in clinical practice, occurring in 1%–4% of the general population (Chinese Society of Cardiac Elect rophysiology and Pacing, 2020). Premature ventricular contractions (PVCs) primarily arise from the outflow tract, with the right ventricular outflow tract being the most frequent source. The mechanism behind PVCs is typically focal, and they exhibit characteristic ECG manifestations (Joshi and Wilber, 2005; Ito et al., 2003; Cronin et al., 2019). Initially, the mapping and ablation of idiopathic RVOT ventricular arrhythmias (VAs) concentrated mainly on areas below the pulmonary valve. In recent decades, with evolving concepts, the mapping scope has expanded to areas above the pulmonary valve, aortic sinus, or aortico-valvular junction regions when managing complex or recurrent cases (Timmermans et al., 2003; Qifang et al., 2019; Dragasis et al., 2022),potentially extending to epicardial regions of the RVOT (Aguilera et al., 2024; Parreira et al., 2019).

Initially, identifying the origin of RVOT premature ventricular contractions depends on surface electrocardiogram (ECG) characteristics, such as R-wave morphology in inferior leads, the precordial transition zone, and ECG-based predictive models (Parreira et al., 2019; Cheng et al., 2018; Di et al., 2019; Penela et al., 2015; Doste et al., 2020), as well as electrophysiological mapping techniques like activation sequence mapping and pace mapping. Although ECG algorithms can locate the regional origin of ventricular arrhythmias (VAs), their spatial resolution is inherently limited, and diagnostic accuracy can be influenced by individual anatomical variations, such as obesity, cardiac rotation, and misplacement of ECG leads (Gabriels et al., 2021),The advent of 3D electroanatomic mapping (EAM) systems (Zhang et al., 2021) has decreased radiation exposure during traditional electrophysiological mapping and enhanced the accuracy of PVC localization; however, discrepancies may still exist between the intended ablation targets and their true anatomical locations.

Recent research by Satoshi Aita and colleagues highlights the clinical potential of combining magnetocardiography (MCG) with cardiac computed tomography (CT) fusion technology (MCG-CT) for localizing RVOT PVC. However, more clinical validation is needed to enhance its application (Aita et al., 2019). Intracardiac echocardiography (ICE), a real-time ultrasound imaging method, provides precise anatomical visualization of cardiac structures, assesses local wall motion abnormalities, and improves catheter tip localization with better contact force monitoring during ablation procedures (Asvestas et al., 2022). Furthermore, ICE has rapidly developed techniques for guiding catheter manipulation, adapting to anatomical variations during radiofrequency ablation (RFA) (Enriquez et al., 2018; Weng et al., 2022).

This study seeks to enhance procedural outcomes for PVC patients. It combines preoperative ECG analysis with intraoperative ICE-guided localization and ablation of PVC origins, which will refine surgical protocols and improve patient prognosis.

## Methods

### Study population

This study consecutively enrolled patients with monomorphic idiopathic ventricular premature contractions admitted to the Sixth Department of Cardiology at the Second Hospital of Hebei Medical University between October 2022 and December 2023. The inclusion criteria required confirmation of the earliest activation site in the right ventricular outflow tract through the CARTO 3D electroanatomical mapping system and intracardiac echocardiography (ICE) during the procedure.

The exclusion criteria were as follows: 1) Patients with organic heart disease, including congenital heart disease, idiopathic cardiomyopathy, or valvular heart disease, or those with poor cardiac function (NYHA functional class > III); 2) Inability to confirm effective ablation targets during the operation or to record ICE data; 3) Incomplete admission or follow-up data regarding standard lead electrocardiograms and 24-h Holter monitoring 3 months after surgery.

All patients received a preoperative evaluation that included a standard 12-lead ECG during PVC episodes, 24-h Holter ECG monitoring, and echocardiography. Radiofrequency ablation procedures were conducted after a washout period of at least five half-lives after stopping antiarrhythmic medications. This study strictly adhered to the principles outlined in the Helsinki Declaration, with written informed consent obtained from all patients. The research protocol received approval from the Research Ethics Committee of the Second Hospital of Hebei Medical University (Ethics Approval Number: 2022-R295).

### Data collection

Upon hospital admission, we collected patients’ baseline information, the count of ventricular premature beats (VPBs) from 24-h Holter ECG recordings, and surface ECG data during VPB episodes. We simultaneously recorded intracardiac electrograms, 3D electroanatomical mapping data, ICE data, and the outcomes of follow-up. All subjects received regular follow-ups at our cardiology outpatient clinic 1 month and 3 months after the procedure, which included 12-lead ECG and 24-h Holter ECG monitoring. We defined recurrence as the reappearance of VPBs with the same morphology, indicating a 24-h burden exceeding 2% in follow-up ECG or Holter examinations.

### Electrocardiogram (ECG) acquisition and measurement

**FIGURE 1:**
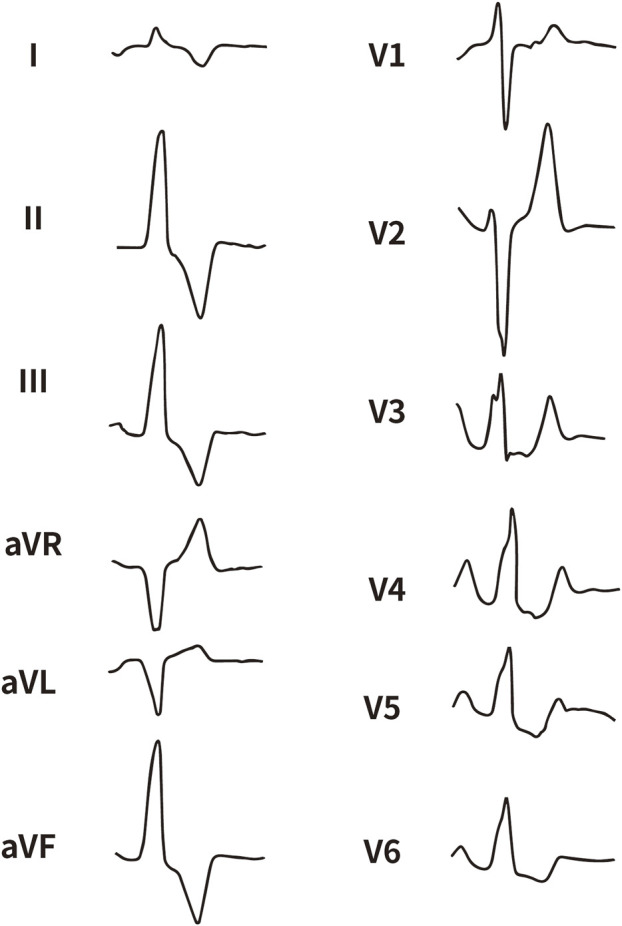
The representative ECG of LPC. LPC, Left pulmonary cusp.

**FIGURE 2:**
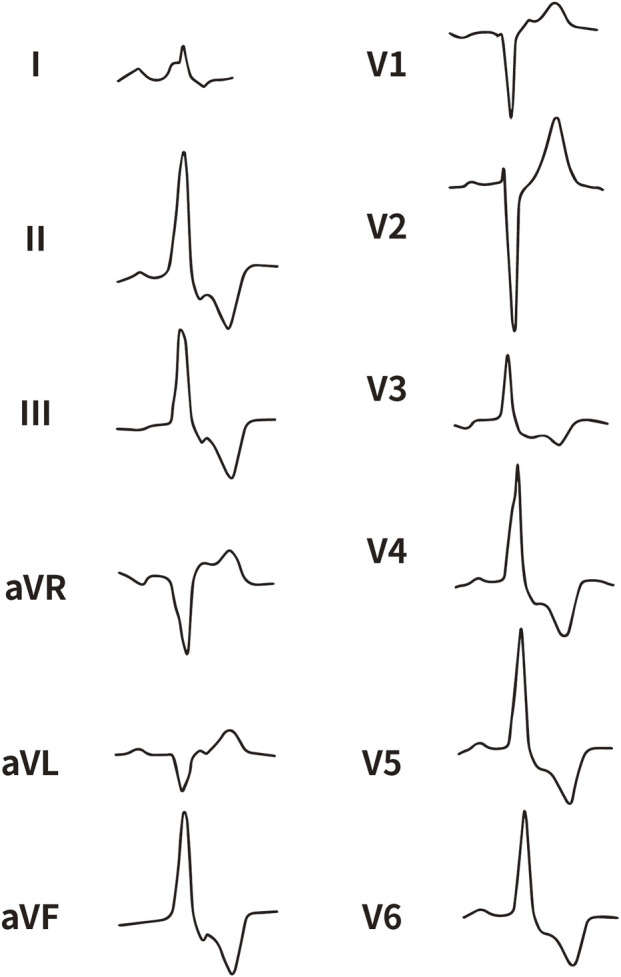
The representative ECG of the junction of LPC and APC. LPC, Left pulmonary cusp; APC, Anterior pulmonary cusp.

**FIGURE 3:**
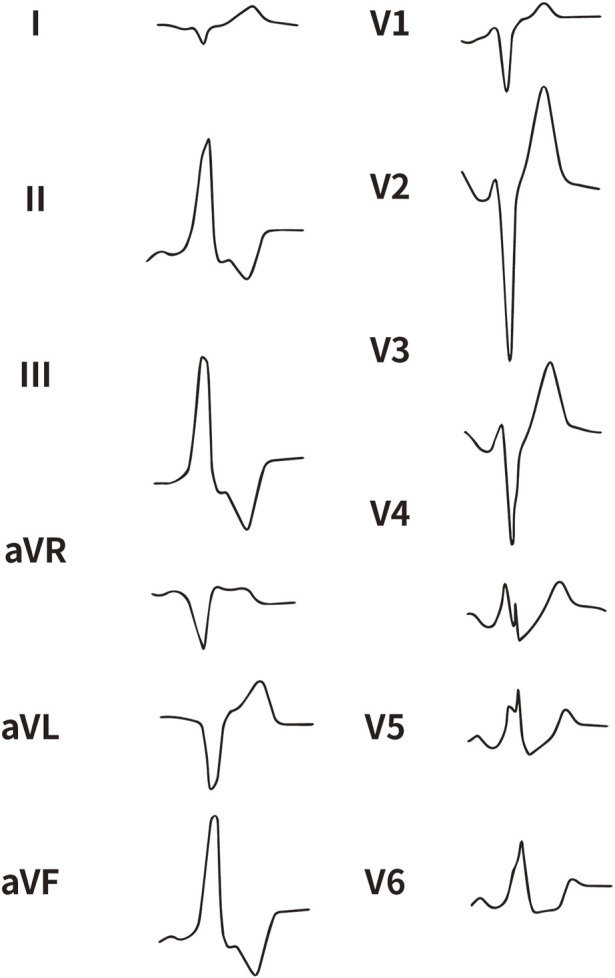
The representative ECG of the junction of RPC and APC. RPC, Right pulmonary cusp; APC, Anterior pulmonary cusp.

**FIGURE 4:**
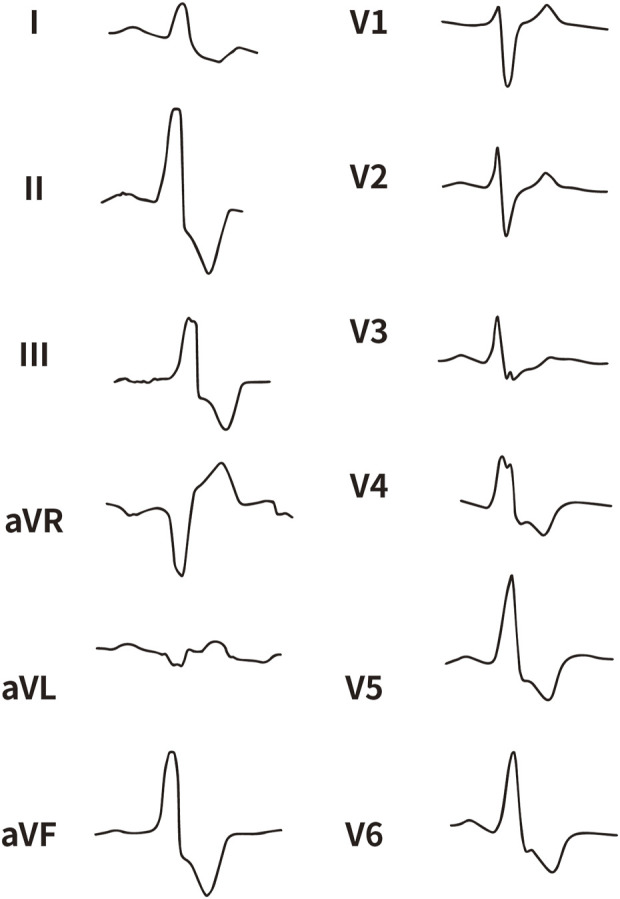
The representative ECG of the junction of LPC and RPC. LPC, Left pulmonary cusp; RPC, Right pulmonary cusp.

All enrolled patients received standard 12-lead ECG recordings during the baseline assessment, using a paper speed of 25 mm/s and a calibration of 10 mm/mV. For all participants, we measured the QRS complex on the R and S waves in the precordial leads (V1, V2, V3) and limb leads (I and aVL), focusing on both amplitude and duration. Amplitude was defined as the vertical distance from the start of the QRS complex to the peak of the R or S wave, while duration was defined as the time interval from the start of the wave’s deflection to its return to baseline or junction with the next waveform. Figures 1–4 illustrate representative ECG patterns of premature ventricular contractions originating from different anatomical sites within the right ventricular outflow tract.

### Application of ICE in operation

**FIGURE 5:**
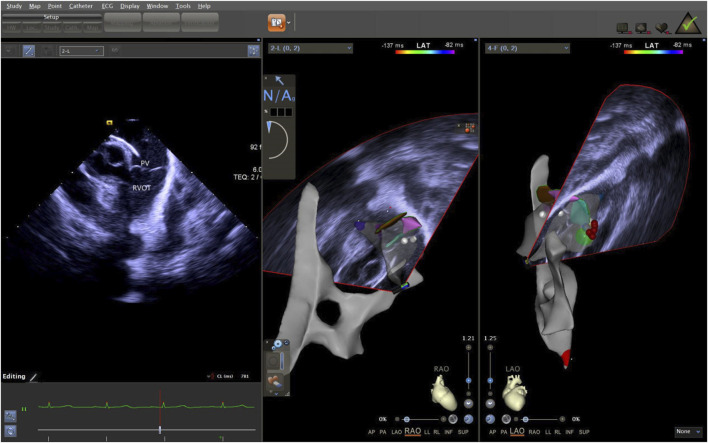
ICE was used to observe the long-axis section of the right ventricular outflow tract. ICE, Intracardiac echocardiography.

**FIGURE 6:**
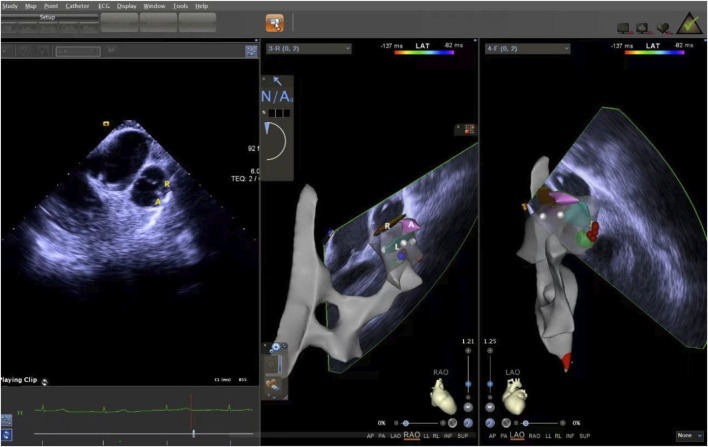
ICE was used to observe the short-axis section of the pulmonary sinus. ICE, Intracardiac echocardiography.

The ICE catheter can be manipulated in multiple directions and angles, allowing for a clear view of all anatomical structures within the cardiac chambers (De Sensi et al., 2021). Advancing the ultrasound catheter with either clockwise or counterclockwise rotation enters the primary field of view of the ICE catheter. This view displays the right atrium (RA), the tricuspid annulus, the aortic sinuses (non-coronary and right coronary), the right ventricular inflow tract, and the outflow tract. By advancing the catheter slightly forward and rotating it clockwise, a short-axis view of the pulmonary artery is achieved. This view reveals the specific positions of the three pulmonary sinuses. In this view, the left pulmonary sinus is closest to the aortic root, while the anterior pulmonary sinus is the farthest from it, positioned superficially next to the pericardium. The right pulmonary sinus is situated slightly to the right of the left pulmonary sinus. Further manipulation of the catheter through the tricuspid annulus into the right ventricle (RV) provides a long-axis view of the pulmonary artery, clearly delineating the RVOT and the pulmonary valve (Figure 5). Collectively, these three views offer a detailed visualization of the pulmonary valve and the three pulmonary sinuses. They also illustrate the junctional regions between the sinuses in the short-axis view (Figure 6).

### Electrophysiological study and radiofrequency ablation procedure

Our research was conducted under a all-zero radiation condition. Radiofrequency ablation was guided by CARTO3 three-dimensional electroanatomical mapping (Biosense Webster, Diamond Bar, CA, United States) and ICE. We used a right femoral venous approach to introduce an 8F sheath. We administered a bolus of 2,000 units of unfractionated heparin intravenously. Next, we advanced a blue saline-irrigated ablation catheter (ThermoCool Smarttouch,ST) through the inferior vena cava into the right atrium. Under ICE guidance, we constructed a point-by-point electroanatomical map. This map delineated the right atrium, tricuspid annulus, right ventricular inflow tract, and RVOT. The catheter was then advanced across the tricuspid annulus into the RVOT.

We performed activation mapping during PVCs to locate the earliest activation site. Optimal mapping targets showed bipolar potentials that either preceded the surface ECG QRS complex by 30 ms or more or exhibited fragmented potentials. Additionally, unipolar electrograms displayed a QS shape. We achieved ablation at sites below the pulmonary valve through direct catheter contact. If we could not adequately reach the target site below the pulmonary valve or if the ablation was ineffective, we performed a ‘reverse U′ maneuver to ablate above the valve, avoiding injury. Consequently, we achieved ablation at sites below the pulmonary valve through direct catheter contact.

**FIGURE 7:**
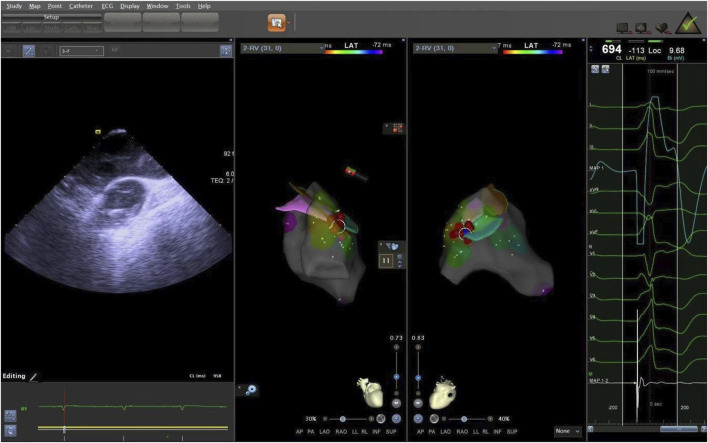
The ablation target of junction of LPC and APC. LPC, Left pulmonary cusp; APC, Anterior pulmonary cusp.

**FIGURE 8:**
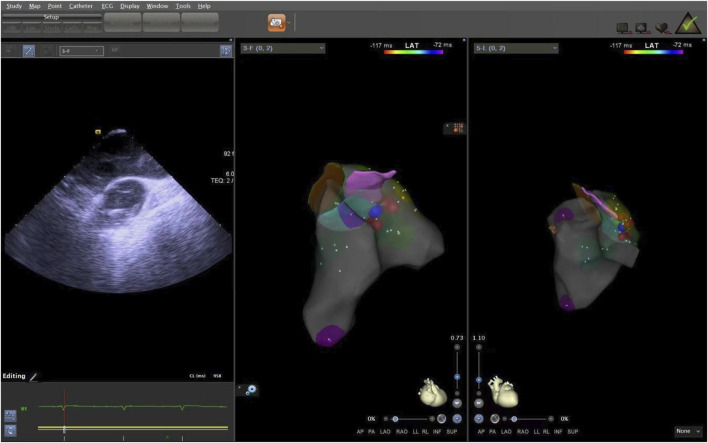
The ablation target of LPC. LPC, Left pulmonary cusp.

The supra-pulmonary sinus position is located above the pulmonary sinus junction plane. The infra-pulmonary sinus position includes locations at or below the pulmonary sinus junction plane, extending within 10 mm from the sinus floor (Acosta et al., 2015). After identifying the ablation target, radiofrequency ablation was performed with preset parameters of 43°C and 30W. The effectiveness of energy delivery (Dragasis et al., 2022) was validated by the disappearance of PVCs during ablation, and the duration of effective ablation was documented. The success criteria were defined as follows: 1) Complete elimination of PVCs on electroanatomic mapping; 2) Absence of preoperative-type premature contractions for 20–30 min after isoproterenol provocation. During the procedure, the CARTO3 three-dimensional anatomical mapping system and ICE were used to record the time, amplitude, and duration of bipolar potentials at the earliest activation site, in relation to the onset of the QRS complex on the surface electrocardiogram. We measured the distance from the effective ablation sites to the pulmonary artery sinus floor. After the procedure, we used ICE again to locate the ablation points, confirming the precise locations of the ablation targets (Figures 7, 8).

### Statistical analysis

All data were analyzed statistically using SPSS 26.0 software (IBM Corp.). Graphs were created using GraphPad Prism 8.0 (GraphPad Software). We assessed the normality of continuous variables using the Shapiro-Wilk (SW) test. Normally distributed data are presented as means ± standard deviations (Mean ± SD). We compared intergroup differences using the independent samples t-test. Non-normally distributed data were reported as median and interquartile range (IQR), with intergroup comparisons performed using the non-parametric Mann-Whitney U test (also known as the Wilcoxon rank-sum test). Categorical variables were analyzed as counts or percentages (%), and we assessed group differences using the Chi-square test. We employed Receiver Operating Characteristic (ROC) curve analysis to calculate sensitivity and specificity, using the area under the curve (AUC) to compare diagnostic accuracy. A two-tailed p-value <0.05 was considered statistically significant.

## Results

### Patient characteristics

**FIGURE 9:**
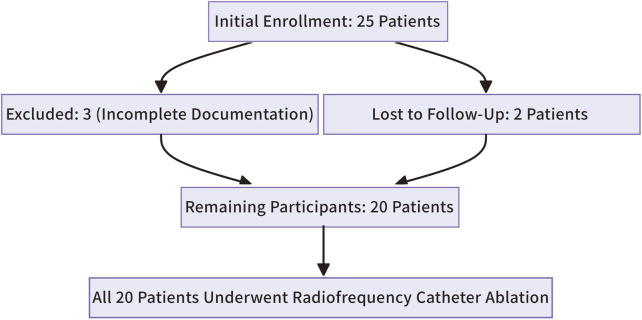
Flow-diagram.

The study initially enrolled 25 patients. Three were excluded due to incomplete clinical or intraoperative documentation, and two were lost to follow-up, leaving 20 participants (Figure 9). All 20 patients successfully underwent radiofrequency catheter ablation, commonly known as RFCA, to treat right ventricular outflow tract ventricular premature beats (RVOT-VPBs). No recurrent ventricular premature beats (VPBs) of the same morphology were observed after administering intravenous isoproterenol at least 20 min post-procedure. Preoperatively, all patients exhibited normal cardiac structure and function, with no evidence of congenital or organic heart disease. Most preoperative clinical manifestations were palpitations, often described as pre-syncope, while a few patients reported chest tightness. Over half of the cohort were female (12 females, 8 males), and most were middle-aged (mean age: 48.4 ± 3.9 years). Baseline characteristics are summarized in Table 1.

**TABLE 1:** Baseline characteristics (N = 20).

Number of patients	20
Age (year)	48.35 ± 3.939
Female	12 (60)
Hypertension	6 (30)
Clinical symptoms	
Palpitations	19 (95)
Palpitations accompanied by other symptoms	5 (5)
Electrocardiographic manifestations	
isolated PVCs	20 (100)
PVCs with VT	0
Prior ablation history	0
Structural heart disease	0
LVEDV (ml)	104.5 (90.75,125.00)
LVEF (%)	63.6 ± 1.57
24-h total PVC count	20815.5 (15410.0,23835.2)

PVCs, premature ventricular contractions; VT, ventricular tachycardia.

LVEDV, Left ventricular end-diastolic volume; LVEF, left ventricular ejection fraction.

### Distribution characteristics of ablation targets under ICE guidance

All 20 patients with RVOT-PVCs successfully underwent RFCA. The ablation sites for the 20 distinct PVCs were unevenly located around the junction of the pulmonary artery and sinus. Of these cases, 3 (15%) were above the sinus, all situated within the left pulmonary sinus, with a mean distance of 3.33 ± 0.33 mm from the base of the left pulmonary sinus. The other 17 cases (85%) were located below the sinus, primarily at three anatomical junctions: 3 cases at the left-right pulmonary sinus junction, 13 cases at the left-anterior sinus junction, and 1 case at the right-anterior sinus junction. The mean distance between sub-sinus ablation targets and the pulmonary sinus base was 2.53 ± 0.38 mm. In a cohort of 20 patients with VPBs where ablation sites were near the pulmonary artery root, the distance from the ablation point to the base of the pulmonary sinus was measured intraoperatively using the CARTO3 three-dimensional mapping system. The patients were categorized into two groups: the supra-pulmonary sinus group (n = 3) and the infra-pulmonary sinus group (n = 17). The analysis showed no statistically significant difference in the measured distances between the two groups (P = 0.394, P > 0.05). Given the higher prevalence of ablation sites located at the junction of the left anterior pulmonary sinus in the infra-pulmonary sinus group, the cohort was further divided into two subgroups: the left-anterior sinus junction group (n = 13) and the non-left-anterior sinus junction group (n = 7). Comparative analysis demonstrated that the ablation sites in the left-anterior sinus junction group were significantly closer to the base of the pulmonary sinus compared to the non-left anterior sinus junction group, with a statistically significant difference (P < 0.05, P = 0.038) (Table 2).

**TABLE 2:** Distance from ablation target to the bottom of pulmonary sinus in botn left anterior sinus junction and non-left anterior sinus junction groups.

Factor	Left anterosinus junctional group (n = 13)	Non-left anterior sinus junction group (n = 7)	Z	*P*
Distance from the effective ablation site to the base of the pulmonary artery sinus (mm)	2.00 (0,3)	3.00 (3,4)	−2.070	0.038

### Ablation methods and ablation duration

All 20 patients with RVOT arrhythmias successfully underwent catheter-based ablation, using either direct catheter apposition at the subvalvular pulmonary valve or the “reverse-U curve” technique at the supravalvular region. Nineteen patients (95%) achieved successful ablation at the subvalvular site, while one patient (5%) required the supravalvular “reverse-U curve” method. The study found that the ablation time was significantly shorter in the subpulmonary sinus group (n = 17) compared to the supravalvular group (P < 0.05, P = 0.013).

### ECG characteristics

Among the 20 patients with RVOT premature ventricular beats, 12 had the transition lead at lead V3 (60%), 5 at lead V2 (25%), and 3 at lead V4. In the 3 cases from the superior pulmonary sinus group, 2 had transition lead at lead V3 (67%), while 1 had it at lead V4 (33%). In the preoperative electrocardiograms of patients in the inferior pulmonary sinus group (n = 17), 10 cases (59%) showed the transition lead at lead V3, while the remaining 7 were at lead V2 and V4. Additionally, there was no significant difference in the transition leads between the two groups, as indicated by a Pearson’s chi-square value of 1.699 (P > 0.05) (Table 3).

**TABLE 3:** Electrocardiographic characteristics of the superior and inferior groups of the pulmonary artery sinus in partial leads.

Leads	Superior group of the pulmonary artery sinus (n = 3)	Inferior group of the pulmonary artery sinus (n = 17)	*P*
Lead V_1_			
R-wave amplitude (mv)	0.40 ± 0.36	0.30 ± 0.21	0.506
S-wave amplitude (mv)	1.30 ± 0.30	1.06 ± 0.48	0.940
R/Sa	0.38 (0.19,0.51)	0.25 (0.16,0.44)	>0.05
R-wave duration (ms)	40.00 ± 40.00	54.12 ± 23.10	0.390
S-wave duration (ms)	90 (60.00,120.00)	80 (60.00,80.00)	0.782
R/Sd	0.67 (0.34,1.00)	0.67 (0.50,1.00)	0.914
Lead V_2_			
R-wave amplitude (mv)	0.36 ± 0.40	0.58 ± 0.39	0.395
S-wave amplitude (mv)	1.77 ± 0.12	1.42 ± 0.80	0.477
R/Sa	0.18 (0.09,0.30)	0.37 (0.14,0.99)	0.289
R-wave duration (ms)	26.67 ± 23.10	61.18 ± 22.05	0.023
S-wave duration (ms)	86.67 ± 30.55	86.47 ± 32.17	0.993
R/Sd	0.50 (0.25,0.59)	0.60 (0.39,1.50)	0.264
Lead V_3_			
R-wave amplitude (mv)	0.70 ± 0.52	0.92 ± 0.49	0.473
S-wave amplitude (mv)	0.60 (0.40,0.80)	0.30 (0.20,0.95)	0.631
R/Sa	1.67 (0.89,3.34)	2.00 (0.37,4.42)	0.958
R-wave duration (ms)	80 (60.00,100.00)	80 (80.00,100.00)	0.771
S-wave duration (ms)	40 (40.00,60.00)	80 (40.00,80.00)	0.612
R/Sd	1.83 ± 1.26	1.25 ± 0.88	0.332
V_2_S/V_3_R	1.90 (1.80,9.45)	2.00 (0.90,3.88)	0.427
Lead aVL			
R-wave amplitude (mv)	0.00 ± 0.00	0.19 ± 0.37	0.420
S-wave amplitude (mv)	0.83 ± 0.06	0.76 ± 0.51	0.802
R/Sa	0.00 ± 0.00	0.76 ± 1.99	0.138
R/Sd	0.00 ± 0.00	0.38 ± 0.34	<0.001
Lead I			
R-wave amplitude (mv)	0.13 ± 0.10	0.42 ± 0.46	0.039
S-wave amplitude (mv)	0.12 ± 0.03	0.24 ± 0.22	0.044
R/Sa	1.28 ± 1.11	1.97 ± 2.06	0.585
R-wave duration (ms)	40.00 ± 20.00	90.59 ± 44.78	0.017
R/Sd	0.83 ± 0.57	0.95 ± 0.58	0.732

**FIGURE 10:**
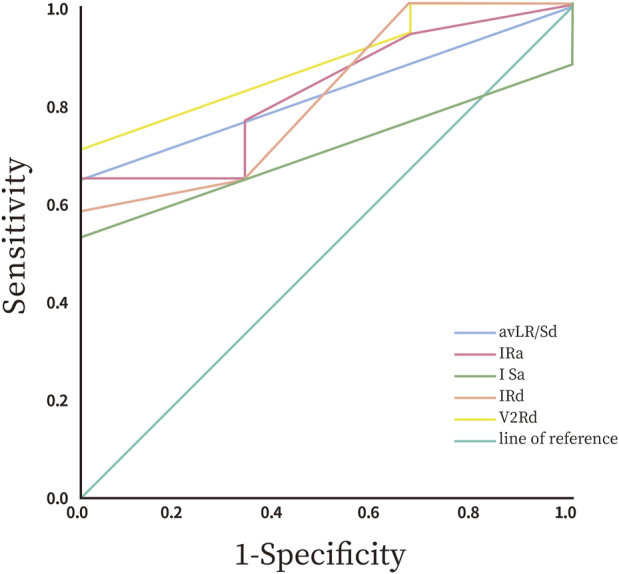
ROC curve of the ECG characteristics.

The study revealed statistically significant differences in preoperative ECG parameters between the supra-pulmonary artery group and the pulmonary sinus group concerning RVOT premature ventricular contraction ablation sites. The specific metrics that differed included R-wave duration in lead V2, the R/S duration ratio in lead aVL, and R-wave and S-wave amplitudes in lead I. The infra-pulmonary artery group showed significantly longer R-wave durations in leads V2 and I, larger S-wave amplitudes in lead I, and smaller R-wave amplitudes compared to the supra-pulmonary artery group (P < 0.05; Table 3). The optimal cut-off value for R-wave duration in lead V2 was 50 ms (AUC = 0.882, 95% confidence interval: 0.718–1.00, P < 0.05). This indicates that an R-wave duration of 50 ms or greater in lead V2 is the predictor for identifying ablation targets in the infra-pulmonary artery region, with a sensitivity of 70.6% and a specificity of 100% (Figure 10; Table 4). There were no significant differences in R-wave or S-wave amplitudes and durations between the two groups in leads V1 and V3 (P > 0.05).

**TABLE 4:** ROC analysis of the ECG characteristics.

Factor	AUC	Standard error	95%CI	P	Cutoff value	Sensitivity	Specificity
R-wave duration in lead V2	0.882	0.084	0.718–1.000	0.039	50	70.6%	100%
R/Sd in lead aVL	0.824	0.097	0.633–1.000	0.081	0.165	64.7%	100%
R-wave amplitude in lead I	0.824	1.107	0.614–1.000	0.081	0.275	64.7%	100%
S-wave amplitude in lead I	0.706	0.115	0.481–0.931	0.266	0.175	52.9%	100%
R-wave duration in lead I	0.814	0.119	0.581–1.000	0.090	70	58.8%	100%

### Intraprocedural complications and postoperative follow-up

Mapping and ablation were performed at target sites near the pulmonary artery root in all patients. After energy delivery, premature ventricular contractions (PVCs) completely resolved. Following intravenous administration of isoproterenol and a 20–30-min observation period, PVCs with the same morphology as pre-procedure did not reappear, yielding an immediate procedural success rate of 100%. No intraprocedural complications—such as other arrhythmias, pericardial tamponade, or thromboembolism—occurred in any patient. All patients underwent scheduled follow-up at 1 month and 3 months post-radiofrequency catheter ablation. At the 1-month follow-up, surface electrocardiograms demonstrated no recurrence of PVCs with the pre-ablation morphology, and 24-h ambulatory electrocardiogram monitoring revealed no PVCs exceeding 10% of total heartbeats. At the 3-month follow-up, no recurrence was observed in any of the 20 patients.

## Discussion

In recent years, ICE has become more widely used in surgeries because it offers real-time visualization of RVOT anatomy, especially in locating ectopic foci near the pulmonary valve (Bartel et al., 2014). The overall complication rate remains below 5%, with severe complications being rare (De Sensi et al., 2021; Lavalle et al., 2020; Pu et al., 2024). Previous studies have found (De Sensi et al., 2021; Liu et al., 2019) that RVOT PVCs usually originate in the peripulmonary valvular regions, particularly within the pulmonary sinuses (PSC), as well as adjacent myocardial tissues. The main anatomical sites include the anterior pulmonary sinus and nearby pulmonary arterial walls, which can be accurately localized using ICE with short-axis and long-axis views of the pulmonary artery, along with three-dimensional mapping. This study involved 20 patients who successfully received ICE-guided radiofrequency ablation for RVOT PVCs. Preprocedural ICE clearly delineated cardiac anatomy in all cases, with ablation sites determined using activation mapping and pace-mapping techniques. All RVOT PVC origins in this cohort were located near pulmonary sinuses, confined within 3.33 mm above and 2.53 mm below the sinus level. The ablation targets for RVOT PVCs predominantly clustered at the left-anterior sinus junction. Moreover, our study was conducted in radiation-free conditions, ensuring complete protection from radiation exposure for both surgeons and patients—a feature unprecedented in previous research.

Numerous global studies have explored methods to determine the origin of PVCs using preoperative ECG analysis. These methods include several novel indices: the Transitional Zone Index (TZ index) for assessing precordial R/S transition patterns (Yoshida et al., 2011), the V2 lead R/S transition rate (Yoshida et al., 2014), and the V2 transition ratio derived from V2 lead waveform characteristics (Betensky et al., 2011). Additionally, the V2S angle algorithm analyzes the orientation of the V2 S-wave vector (Qiu et al., 2022), while R-wave amplitude in lead I (Wang et al., 2021), the Initial R-wave surface area index (ISA) in V1/V2 (Zhang et al., 2023), and the composite value of [(V1S + V2S) - (V1R + V2R)] (Kaypakli et al., 2018) are employed to differentiate PVCs originating from the RVOT and the left ventricular outflow tract (LVOT). The composite value of [(V1S + V2S) - (V1R + V2R)] has a cutoff threshold of 1.625 mV for predicting PVCs originating from the RVOT, achieving a sensitivity of 95.1% and specificity of 85.5%. Some studies have integrated the V2 transition ratio with the V2S/V3R index—defined as the ratio of S-wave amplitude in lead V2 to R-wave amplitude in lead V3—to develop a comprehensive diagnostic model represented by Y = −1.15 × TZ index - 0.494 × V2S/V3R. This model achieved an AUC of 0.88, significantly outperforming single parameters (He et al., 2018). However, research on distinguishing premature ventricular contractions (PVCs) from specific sub-regions within the RVOT is still limited. Our findings provide a novel insight by demonstrating that an R-wave duration of 50 milliseconds or greater in lead V2 serves as a predictor for sub-pulmonary cusp PVCs, achieving a sensitivity of 70.6% and a specificity of 100%.

RFCA is now the primary treatment for symptomatic RVOT-PVCs, especially in patients who do not respond to medication, have a high PVC burden (≥10% on 24-h Holter monitoring), or also suffer from left ventricular dysfunction (Lavalle et al., 2020; Wojdyła-Hordyńska et al., 2020). This approach demonstrates high efficacy (acute success rates of 80%–90% reported in literature) and favorable safety profiles (Lavalle et al., 2020; Jiang et al., 2024). Early studies showed that traditional methods using 12-lead surface ECG localization, fluoroscopy, and intracardiac electrophysiological mapping had success rates of 80%–85% for RVOT-PVC ablation. (Jiang et al., 2024; Ye et al., 2022; Chaumont et al., 2023). In this study, we aimed to evaluate the predictive value of surface electrocardiograms (ECGs) for identifying PVCs originating from the right ventricular outflow tract (RVOT) and to assess the utility of intracardiac echocardiography (ICE) in facilitating their radiofrequency ablation. We conducted a prospective analysis involving 20 patients with RVOT-PVCs, employing ICE and CARTO 3D electroanatomical mapping to classify ablation sites and analyze preoperative ECG parameters. Our findings revealed a clear anatomical distinction in PVC origins. This underscores the importance of integrating advanced imaging techniques and ECG analysis in the management of PVCs, optimizing ablation strategies, and potentially improving patient outcomes.

The use of new technologies, such as advanced irrigated contact-force ablation catheters (e.g., Thermocool SmartTouch) (Zhao et al., 2020), very high-power short-duration (vHP-SD) ablation protocols (Heeger et al., 2022a; Heeger et al., 2022b), and ablation index-guided (AI) strategies (Gasperetti et al., 2021),has increased success rates to over 90%, with some studies reporting rates as high as 95%. Additionally, using 3D mapping systems has greatly enhanced procedural success, especially in anatomically complex regions of the RVOT (Zhang et al., 2021; Chaumont et al., 2023; Rodkiewicz et al., 2024; Letsas et al., 2019). In terms of recurrence rates, intermediate-term follow-up indicates that recurrences occur in 5%–15% of cases, which correlates with the complexity of the anatomy and the completeness of the mapping (Chaumont et al., 2023; Chen et al., 2022). In this study, the integration of conventional ECG localization with contact-force catheters and 3D electroanatomical mapping achieved 100% acute procedural success, with no recurrences observed at 3-month follow-up, thereby validating the therapeutic effectiveness of this approach.

## Conclusion

This study conducted zero-fluoroscopy ablation for idiopathic PVCs, guided by electrocardiography, three-dimensional electroanatomical mapping, and ICE. The key conclusions are as follows.

1. Preoperative ECG demonstrates certain predictive value for identifying the origin of ventricular premature beats. In lead V2, an R wave duration of 50 milliseconds or longer exhibits certain predictive value for ventricular premature beats originating below the pulmonary valve sinus.
2. ICE can clearly visualize the anatomical structures of the right ventricular outflow tract, which aids in accurately determining the ablation site under all-zero fluoroscopy.
3. The origin of PVCs in RVOT is typically located near the pulmonary sinus cusps. For PVCs originating below the pulmonary valve, the ablation target sites are primarily concentrated at the junction of the left-anterior pulmonary sinus cusp.

## Limitations

This study has several limitations. First, it is a single-center, prospective, exploratory study that is non-controlled, has a small sample size, and features a short follow-up duration. In our study group, 85% of the ablation sites were positioned below the junction of the pulmonary sinus cusps. Due to the anatomical constraints and limitations in catheter positioning, only one case applied the “reverse-U″ ablation technique, while the other 17 cases used conventional catheter contact ablation in the subvalvular region. This distribution may have hindered a proper comparison of the potential advantages between the reverse-U technique and direct catheter contact ablation. Second, as this study exclusively included consecutive patients undergoing ICE-assisted RVOT PVC ablation, we lacked a control group of patients undergoing conventional RVOT PVC ablation without ICE guidance, thereby precluding direct comparative analysis. Finally, the relatively small sample size of 20 patients, though sufficient to draw preliminary conclusions, may limit the generalizability of our findings to broader patient populations. Future studies should aim to include a larger, multicentric cohort to validate our findings and explore the long-term outcomes associated with the identified predictive ECG parameters.

## Data Availability

The original contributions presented in the study are included in the article/supplementary material, further inquiries can be directed to the corresponding author.
